# The molecular dimension of microbial species: 3. Comparative genomics of *Synechococcus* strains with different light responses and *in situ* diel transcription patterns of associated putative ecotypes in the Mushroom Spring microbial mat

**DOI:** 10.3389/fmicb.2015.00604

**Published:** 2015-06-23

**Authors:** Millie T. Olsen, Shane Nowack, Jason M. Wood, Eric D. Becraft, Kurt LaButti, Anna Lipzen, Joel Martin, Wendy S. Schackwitz, Douglas B. Rusch, Frederick M. Cohan, Donald A. Bryant, David M. Ward

**Affiliations:** ^1^Department of Land Resources and Environmental Sciences, Montana State UniversityBozeman, MT, USA; ^2^Department of Mathematical Sciences, Montana State UniversityBozeman, MT, USA; ^3^Department of Energy, Joint Genome InstituteWalnut Creek, CA, USA; ^4^J. Craig Venter InstituteRockville, MD, USA; ^5^Department of Biology, Wesleyan UniversityMiddletown, CT, USA; ^6^Department of Biochemistry and Molecular Biology, The Pennsylvania State UniversityUniversity Park, PA, USA; ^7^Department of Chemistry and Biochemistry, Montana State UniversityBozeman, MT, USA

**Keywords:** comparative genomics, microbial species, thermophilic *Synechococcus*, microbial mats, adaptation

## Abstract

Genomes were obtained for three closely related strains of *Synechococcus* that are representative of putative ecotypes (PEs) that predominate at different depths in the 1 mm-thick, upper-green layer in the 60°C mat of Mushroom Spring, Yellowstone National Park, and exhibit different light adaptation and acclimation responses. The genomes were compared to the published genome of a previously obtained, closely related strain from a neighboring spring, and differences in both gene content and orthologous gene alleles between high-light-adapted and low-light-adapted strains were identified. Evidence of genetic differences that relate to adaptation to light intensity and/or quality, CO_2_uptake, nitrogen metabolism, organic carbon metabolism, and uptake of other nutrients were found between strains of the different putative ecotypes. *In situ* diel transcription patterns of genes, including genes unique to either low-light-adapted or high-light-adapted strains and different alleles of an orthologous photosystem gene, revealed that expression is fine-tuned to the different light environments experienced by ecotypes prevalent at various depths in the mat. This study suggests that strains of closely related PEs have different genomic adaptations that enable them to inhabit distinct ecological niches while living in close proximity within a microbial community.

## Introduction

Thermophilic cyanobacteria of the genus *Synechococcus* predominate in microbial mat communities inhabiting the effluent channels of alkaline, siliceous hot springs and have been extensively studied and characterized for over 50 years (Peary and Castenholz, 1964; Brock, 1978; Ward et al., 2012). Strains of *Synechococcus* from mats in Hunters Hot Springs, OR, which were found to exhibit different temperature adaptations, were first observed by Peary and Castenholz (1964). Molecular analyses of Octopus Spring, Yellowstone National Park (YNP), based on 16S rRNA sequences provided further evidence of *Synechococcus* ecotypes—five closely related genotypes were found to be distributed differently from high to low temperatures along the thermal gradient of the effluent channel (Ferris and Ward, 1997). Genotype A” inhabited the highest temperatures, followed by A′, A, B′, and B as temperatures decreased with distance from the source, though some overlap of adjacent genotypes was observed. Strains with these 16S rRNA genotypes were shown to have different temperature adaptations that correlated with their distribution along the effluent channel (Allewalt et al., 2006). 16S rRNA genotypes were also found to differ along the vertical aspect of the mat. For example, differential vertical distributions of genotype B′, which occurred above genotype A in the 60°C mat, were observed (Ramsing et al., 2000). However, fluorescence microscopy, combined with estimates of oxygenic photosynthetic rates calculated using oxygen microsensor measurements in a 68°C mat sample, revealed physiologically distinct populations in the lower and upper parts of the top green mat layer. These populations were identical at the 16S rRNA locus and might have been interpreted as one genotype acclimated differently in response to lower light intensity. However, surface and subsurface populations were genetically distinct at the more rapidly evolving internal transcribed spacer locus that separates the 16S and the 23S rRNA genes (Ferris et al., 2003), suggesting the possible existence of yet more closely related *Synechococcus* populations with different adaptations to light.

Based on these observations, Ward and Cohan (2005) and Ward et al. (2006) foresaw the need for theory-based models to predict putative ecological species, or ecotypes, from natural variation in sequence data, and for studying genes with even higher molecular resolution than 16S rRNA and the 16S–23S rRNA internal transcribed spacer region. Most recently, *Synechococcus* putative ecotypes (PEs) have been demarcated using highly resolving, protein-encoding loci, from which the evolutionary simulation algorithm Ecotype Simulation (Koeppel et al., 2008) has predicted an even greater number of PEs than demarcated by the internal transcribed spacer region (Becraft et al., 2011; Melendrez et al., 2011). As an example, in the first paper of this three-paper series on the molecular dimensions of microbial species, Becraft et al. (2015) used *psaA* sequence variation and Ecotype Simulation to predict many PEs, including seven PEs in the A′ lineage, 15 PEs in the A lineage, and 24 PEs in the B′ lineage, several of which were shown to have different vertical distributions in the mat. At 60–63°C, PEs B′9, A1, A4, A14, and A6 were found to be progressively predominant from the mat surface to the bottom of the upper 1 mm-thick green layer (summarized in Table 1; also see Figure 3 in Becraft et al., 2015). This led to the hypothesis that these PEs are adapted to different irradiances corresponding the light levels they experience *in situ*. In the second paper of the series, Nowack et al. (2015) reported the successful cultivation of strains representative of PEs A1, A4, and A14, which they used to test the hypothesis. Strains representative of these PEs were shown to have distinctive growth patterns, pigment contents, and low-temperature fluorescence emission spectra when grown at either high or low irradiance. These differences indicated adaptive and/or acclimative responses to irradiance levels and light qualities that are characteristic of the depth at which each PE predominates *in situ* (see Figures 4, 8C in Becraft et al., 2015).

**Table 1:** **Genomic, phenotypic, and environmental information for *Synechococcus* strains of putative ecotypes with different depth distributions^a^ or from different hot spring mats**.

**Strain**	**A4**	**A14**	**A1-MS**	**A1-OS**
Strain name	65AY6A5	60AY4M2	65AY6Li	JA-3-3Ab
Putative ecotype (PE)	A4	A14	A1	A1
Collection source	Mushroom Spring	Mushroom Spring	Mushroom Spring	Octopus Spring
Isolation date	September 2010	September 2010	September 2010	July 2002
Isolation temperature (°C)	65	60	65	58–65^b^
PE relative abundance ≥5% at 60°C Mushroom Spring	400–800 μm	400–960 μm	0–960 μm	0–960 μm
PE relative abundance ≥10% at 60°C Mushroom Spring	640–720 μm	560–960 μm	0–800 μm	0–800 μm
Light adaptation	Low-light	Low-light	High-light	High-light
Sequencing method	Illumina	Illumina	Illumina	Sanger
Depth of coverage	22×	35×	16×	2×
Length (Mbp)	2.98	3.16	2.93	2.93
Number of contigs	9	6	2	1
Largest contig (bp)	2,508,234	3,142,301	2,795,989	2,932,766
%GC content	60.4	60.4	60.3	60.2
CDS	2622	2597	2623	2760
tRNAs	47	47	49^c^	47
rRNA operons	2	2	2	2
Number of genes unique to the strain with respect to the reference A1-OS	173	204	131	N/A

a *Distributions are expressed as a range of depths where the PE has relative abundance of either ≥5 or ≥10% abundance, rather than emphasizing the peak population abundance of a PE (e.g., Table 1 in Nowack et al., 2015)*.

b *Temperatures in Octopus Spring fluctuate continuously over a 4.5 min cycle (Miller et al., 1998), therefore the 7°C range of the isolation site is given*.

c *A1-MS contains a duplication of the 23S rRNA locus in one operon and two adjacent tRNA loci in one operon*.

While individual genes can be used to predict ecotypes whose unique niches have been inferred from their microhabitat distributions (Becraft et al., 2015), and whose existence can be confirmed by the phenotypes of strains (Nowack et al., 2015), whole genome comparative analysis can reveal genetic differences among strains that may be responsible for the adaptive and acclimative mechanisms. For example, the genomes of closely related strains of *Prochlorococcus* spp., which are prevalent phototrophs in marine environments, have been sequenced and compared. Strains that have different light adaptations maintain differences in gene content related to adaptation to the specific light and nutrient environment of the surface-associated high-light or deep-water-associated low-light layers in the ocean (Rocap et al., 2003). *Prochlorococcus* spp. strains with similar light adaptations also maintained “genomic islands” that may aid in niche differentiation of ecotypes that co-exist within the high-light or the low-light portions of the water column (Coleman et al., 2006; Kettler et al., 2007). Furthermore, genomic analyses are open-ended and unconstrained by the limits of our intuition in that they may reveal unsuspected differences for physiological or metabolic functions that have not yet been tested experimentally. For instance, in previous work the genome of a *Synechococcus* genotype found in downstream regions of these hot spring mats (i.e., a B′-like strain) was shown to have genes for nitrogen storage and metabolism and for phosphonate utilization that were lacking in an upstream genotype (i.e., A-like), indicating that populations along the flow path differ in adaptations for nutrient metabolism as well as temperature (Bhaya et al., 2007).

In this study, we compared the genomes of four *Synechococcus* strains within the A lineage that are representative of PEs known to be predominant at different depths in the 60–63°C Mushroom Spring mat (Becraft et al., 2015). Strains representative of PE A1, which is found closer to the mat surface, and PEs A4 and A14, which are found deeper in the mat upper green layer, were shown to have different adaptations and acclimative behaviors to low and high irradiance (Nowack et al., 2015). A second PE A1 strain, which had been previously cultivated from Octopus Spring (Allewalt et al., 2006) was shown to have light responses that were indistinguishable from that of the other PE A1 strain (Nowack, 2014). We compared the genomes of these strains of high-light and low-light adapted organisms to identify differences in gene content and specific alleles that might underlie these and other adaptations and acclimative responses. We also used these genomes to probe transcript abundances for specific ecotypes using a diel metatranscriptome dataset we had previously obtained (Liu et al., 2012). We sought evidence of differences in transcription patterns for homologous genes shared among species, which were divergent enough to differentiate PEs, as well as strain-specific genes, which may be representative of each PE, including genes involved in light harvesting and nutrient uptake. These differences may be indicative of mechanisms underlying adaptive and acclimative responses to light intensity, light quality, and nutrient use that reflect the distinct, ecological niches of these PEs.

## Materials and methods

### *Synechococcus* strains

Strains representative of *Synechococcus* PE A1 (65AY6Li), PE A4 (65AY6A5), and PE A14 (60AY4M2), were selected for comparative genomic analysis. For simplicity we will refer to these strains by their PE affiliations or by their known adaptive and/or acclimative responses to low light (strains of PEs A4 and A14) or high light (strains of PE A1). The DNA samples used to demonstrate strain purity by Ti-454 barcode sequencing (Nowack et al., 2015) were also used for genome sequencing. These genomes were compared to the genome of a second strain of PE A1 (JA-3-3Ab), previously obtained from the microbial mat of Octopus Spring (Bhaya et al., 2007), which was also shown to be adapted to high light (Nowack, 2014). To distinguish between these two PE A1 strains, we will refer to them here as strain PE A1-MS (from Mushroom Spring) and PE A1-OS (from Octopus Spring). Mushroom Spring is located ~0.5 km from Octopus Spring, and the two alkaline siliceous springs have been shown to have similar major ion chemistry over decades (see Brock, 1978; Inskeep et al., 2013; the YNP Research Coordination Network website^1^) and inhabitants (Ramsing et al., 2000; Becraft, 2014).

### Genome sequencing, assembly, and annotation

Purified total genomic DNA from each strain was submitted to the Department of Energy Joint Genome Institute for sequencing and assembly. The DNA from each strain was randomly sheared into ~270 bp fragments and the resulting fragments were used to create fragment libraries. These libraries were sequenced on Illumina sequencers generating 150-bp paired-end reads. All general aspects of library construction and sequencing are described on the JGI website^2^. Because the variant detection pipeline requires some non-overlapping paired reads prior to variant detection, the reads were then trimmed to 125 bp. These trimmed reads were then aligned to the reference genome *Synechococcus* sp. JA-3-3Ab using the Burrows-Wheeler Aligner (BWA) (Li and Durbin, 2009), and putative single-nucleotide polymorphisms (SNPs) and small indels were identified using samtools and mpileup (Li et al., 2009). Putative structural variants were identified using BreakDancer (Chen et al., 2009), filtering for a confidence score of >90. Genomes were also assembled *de novo* for each strain. Each FASTQ file was QC-filtered for artifact/process contamination and subsequently assembled with AllPathsLG (Gnerre et al., 2011). The resulting contigs included DNA sequences of heterotrophic contaminants that occur in these strains (Nowack et al., 2015). These sequences were separated from *Synechococcus* sequences by binning the sequences using NCBI BLASTN and a database of the *Synechococcus* spp. JA-3-3Ab and JA 2-3B′a(2-13) reference genomes (Bhaya et al., 2007), and those of the possible contaminants (*Meiothermus* spp., CP005385; *Anyoxybacillus* spp., CP000922; *Rubrobacter* spp., CP000386) using a method similar to that described in Klatt et al. (2011). The *Synechococcus* DNA assemblies were submitted to the automatic annotation pipelines NCBI PGAAP [NCBI Handbook (Internet) 2nd edition (Tatusova et al., 2013)] and RAST (Aziz et al., 2008) for annotation using the default parameters. The three draft genome sequences have been submitted to Genbank under the following accession numbers: PRJNA209725 (*Synechococcus* sp. 65AY6Li, PE A1), PRJNA210217 (*Synechococcus* sp. 65AY6A5, PE A4), and PRJNA210214 (*Synechococcus* sp. 60AY4M2, PE A14).

### Comparative analyses

The strain genome phylogeny was obtained using a concatenation of 460 marker proteins identified by Phyla-AMPHORA (Wang and Wu, 2013), which uses phylum-specific conserved proteins for metagenomic phylotyping. Only conserved cyanobacterial proteins present in all five genomes (the strains studied here and *Synechococcus* sp. JA-2-3B′a(2-13), which was used as an outgroup) were selected (highlighted in Supplementary Table S1). Proteins were aligned with ClustalO (Sievers et al., 2011) and a Newick tree was computed using FastTree, with local support values calculated using the Shimodaira_Hasegawa test (Price et al., 2009). Comparative analyses of genomes were conducted using the RAST SEEDViewer for gene content analyses (Overbeek et al., 2014) and the best-hit and reciprocal best-hit average nucleotide identity (ANI) calculator (Goris et al., 2007).

### Metatranscriptomic analyses

Diel metatranscriptomic datasets described by Liu et al. (2012), which were based on analysis of pooled triplicate samples from the Mushroom Spring 60°C mat, collected at hourly intervals throughout a complete diel cycle, were reanalyzed by BWA (Li and Durbin, 2009) to locate transcripts for specific genes. Genes targeted in these analyses were chosen either because they are specific to low-light- or high-light-adapted strains or, in the case of orthologous genes, because they exhibited at least 3% nucleotide sequence difference between/among homologous genes in different strains. We used the methods described in Liu et al. (2011, 2012), except that (i) we used genomes instead of metagenomic assemblies to recruit transcripts, and (ii) transcripts associated with the unique alleles of different ecotypes were recruited without allowing any mismatches (i.e., an exact sequence match was required). Recruitment of transcripts associated with B′-like *Synechococcus* was done using the published *Synechococcus* B′ genome (Bhaya et al., 2007), but, as in Liu et al. (2012), up to 5 mismatches were allowed. This was done because this genome is not representative of the predominant B′ PE (B′9) in the 60°C mat (Becraft et al., 2015), which we do not yet have in culture (Nowack et al., 2015). Raw transcript counts were normalized by the total number of mRNA-specific transcripts at each time point and then by the geometric mean of normalized transcript counts across all time points (Liu et al., 2011, 2012).

## Results

### Genomic properties

Basic characteristics of the genomes of strains representative of PEs A1-OS, A1-MS, A4, and A14 are presented in Table 1. The strains are 99.93–100% identical at the 16S rRNA locus and share 2201 orthologous genes as their core genome, including most RAST annotated subsystem genes found in the A1-OS reference genome. The ANI among orthologous genes in the different strains ranged from 98.35 to 99.32%, so many of the shared genes predict proteins with 100% sequence identity and are likely to be functionally identical (Table 2).

**Table 2:** **Average nucleotide index (ANI) and genes with identical sequence in *Synechococcus* strain genomes**.

	**A4**	**A14**	**A1-MS**	**A1-OS**
A4		42.65%	37.65%	36.63%
A14	99.05%		37.08%	43.77%
A1-MS	98.41%	98.72%		55.07%
A1-OS	98.42%	98.35%	99.32%	

*The ANI between each pair of genomes, using whole-genome reciprocal best hits is shown below the diagonal. The percentage of genes that encode for proteins with identical amino acid sequences (and may be functionally identical) between each pair of genomes is shown above the diagonal*.

**Figure 1:**
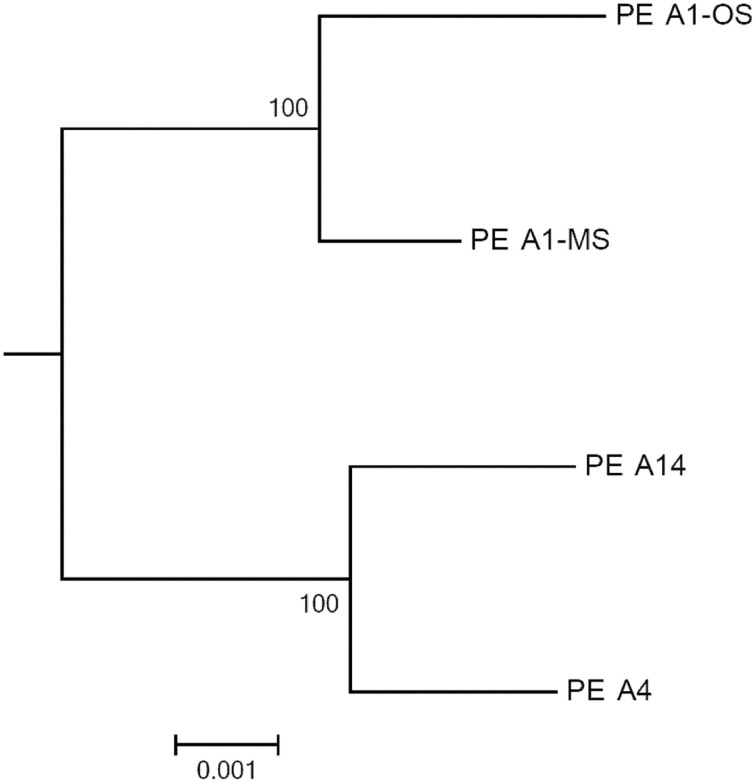
**Phylogeny based on a concatenation of 460 conserved cyanobacterial proteins found in the A-lineage strains**. The tree was rooted by *Synechococcus* sp. JA-2-3B′a(2-13) and the scale bar is equal to 0.001 amino acid substitutions/site.

The GC contents of the strains ranged from 60.2 to 60.4%, while genome sizes varied from 2.93 Mbp for strains of PEs A1-OS and A1-MS to 3.16 Mbp for the PE A14 strain (Table 1). The strain genome phylogeny reflects the *psaA* phylogeny (see Figure 1 in Becraft et al., 2015), with the high-light-adapted strains and the low-light-adapted strains each forming a distinct clade (Figure 1). Although all sequenced A-lineage strains are very closely related, there are several genomic differences among the strains that may underlie the niche differentiation among the PEs. The three strains were selected because they are representative of PEs that differ in vertical position and exhibit different adaptations to irradiance, but the low-light-adapted strains of PEs A4 and A14 were similar to each other phenotypically (see Nowack et al., 2015) and were very different from the two high-light-adapted strains A1-MS and A1-OS. Hence, this discussion will focus on differences between the two low-light-adapted strains and the two high-light-adapted strains. Differences discussed in the main text, including any subsystem genes missing in the newly sequenced strains, are presented in Table 3 and a full ortholog table, which also presents differences in the percentage amino acid identity of homologous genes, is presented in Supplementary Table S1. Specific differences between the genomes of PE A4 and A14 strains will also be considered below.

**Table 3:** **Ortholog table showing discussed gene content differences among strains with different light adaptations and representative of putative ecotypes with different vertical positioning in the 60–63°C Mushroom Spring mat**.

**A4**	**A14**	**A1-MS**	**A1-OS**
Chlorophyll a(b) binding protein, photosystem II CP43 protein homolog *isiX*/ Allophycocyanin subunit *apcB3*/ Allophycocyanin subunit *apcD4*/Hypothetical protein (putative photoreceptor)	Chlorophyll a(b) binding protein, photosystem II CP43 protein homolog *isiX*/ Allophycocyanin subunit *apcB3*/ Allophycocyanin subunit *apcD4*/ Hypothetical protein (putative photoreceptor)		
Ferrous iron transport cassette *feoAB* (2 genes)	Ferrous iron transport cassette *feoAB* (2 genes)		
Maltose/maltodextrin transport cluster *malK* (4 genes)	Maltose/maltodextrin transport cluster *malK* (4 genes)		
Ammonium transporter *amtB2*	Ammonium transporter *amtB2*		
Ammonium transporter *amtB1*	Ammonium transporter *amtB1*	Ammonium transporter *amtB1*	Ammonium transporter *amtB1*
Assimilatory nitrate reductase	Assimilatory nitrate reductase	Assimilatory nitrate reductase (inactivated)	Assimilatory nitrate reductase (inactivated)
Methyl-accepting chemotaxis protein	Methyl-accepting chemotaxis protein	Methyl-accepting chemotaxis protein (inactivated)	Methyl-accepting chemotaxis protein (inactivated)
		Carbonic Anhydrase	Carbonic Anhydrase
		Peptide/opine/ nickel PepT ABC Transport Cassette (5 genes)	Peptide/opine/ nickel PepT ABC Transport Cassette (5 genes)
		Urease Cluster 1 (5 genes)	Urease Cluster 1 (5 genes)
		Cystine ABC Transporter (2 genes)	Cystine ABC Transporter (2 genes)
		Succinate dehydrogenase flavoprotein subunit *sdhA*/ Omega-amino acid-pyruvate amino-transferase	Succinate dehydrogenase flavoprotein subunit *sdhA*/ Omega-amino acid-pyruvate amino-transferase
		Type III CRISPR/*cas* array	Type III CRISPR/*cas* array
Urea Carboxylase Cassette (7 genes)	Urea Carboxylase Cassette (7 genes)	Urea Carboxylase Cassette (7 genes)	
Polar amino acid ABC Transport (PAAT) (3 genes)		Polar amino acid ABC Transport (PAAT) (3 genes)	Polar amino acid ABC Transport (PAAT) (3 genes)
	Sugar ABC Transport Cassette (3 genes)	Sugar ABC Transport Cassette (3 genes)	Sugar ABC Transport Cassette (3 genes)
Spermidine Putrescine ABC Transporter PotABCD (6 genes)			
Beta-carotene ketolase			
Formamidase *amiF*/Amidase Gene Cluster (8 genes)			
		Type I restriction-modification system/SS exonuclease associated with Rad50/Mre11 complex/Bipolar DNA Helicase	

### Genes found only in low-light-adapted strains and their diel transcription patterns

The low-light-adapted strains representative of PEs A4 and A14 have a unique, possibly horizontally acquired gene cluster that includes: a potential photoreceptor predicted to have four PAS domains, two GAF domains, and a histidine kinase domain, which could act as a light-activated response regulator; *apcD4* and *apcB3* genes, which are predicted to encode a variant allophycocyanin that probably has enhanced far-red absorption (Gan et al., 2014b); and a gene for an IsiA-like protein, which we have tentatively named IsiX. Allophycocyanins are phycobiliproteins that absorb red and far-red light and form light-harvesting antenna complexes for Photosystems I and II in cyanobacteria (Gan et al., 2014a,b; Sidler, 2004). ApcD4 is approximately 40% identical and 62% similar to ApcD1 (CYA_2790) in these *Synechococcus* strains and is ~63% similar to ApcA (CYA_2227) of the PE A1-OS strain; ApcB3 is ~80% similar to the ApcB (CYA_2226) of the PE A1-OS strain. In contrast, the products of the *apcA* and *apcB* genes, which are not located in this cluster, are highly conserved in all four strain genomes (100% identical). ApcD4 and ApcB3 are only found in a few cyanobacteria and probably form a variant type of allophycocyanin with enhanced far-red light absorption (670–710 nm absorption maximum) (Gan et al., 2014b).

IsiA and its paralogs are chlorophyll-binding proteins that form specialized light-harvesting antenna complexes in cyanobacteria (Kouril et al., 2005; Murray et al., 2006). IsiX belongs to the PsbC/IsiA superfamily (Kouril et al., 2005; Murray et al., 2006) of chlorophyll (Chl) *a*-binding antenna complex proteins but is quite distinct from IsiA, which typically produces a specialized light-harvesting complex under iron-starvation conditions (Table 3). IsiX, which has a C-terminal extension of nearly 100 amino acids and probably has one additional transmembrane helix relative to other PsbC/IsiA proteins, is only 48% similar to the paralogous Photosystem II core subunit, PsbC, which is >99% similar among all four strains. Moreover, IsiX is only 46% similar to IsiA (CYA_2606), which is likely to be iron-regulated because of its co-localization with *isiB*, encoding flavodoxin (IsiB; CYA_2605), as observed in most other cyanobacteria (Straus, 1994). These observations are consistent with the idea that ApcD4-ApcB3 and IsiX are specialized antenna proteins that function in PE A4 and A14 strains under low irradiance or possibly far-red light (or both) conditions. Consistent with this idea, we have noted that strains corresponding to PEs A4 and A14 have enhanced absorption above 700 nm compared to the PE A1-MS strain (data not shown, but see Nowack et al., 2015). However, at this point it is not yet clearly established whether these proteins are linked to these differences.

**Figure 2:**
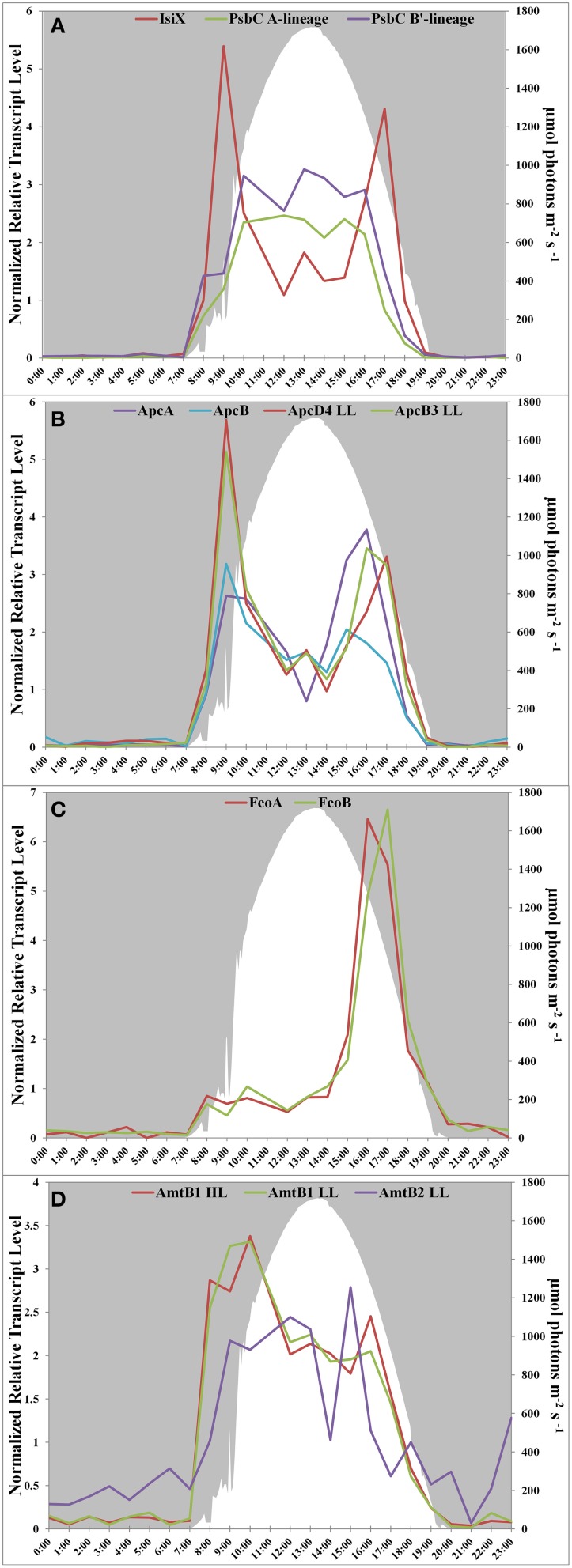
**Transcription patterns of transcripts encoding (A) PsbC in all A-lineage strains and IsiX, present in PE A4 and A14 strains, (B) ApcA/ApcB, present in all A-lineage strains, and ApcD4 LL/ApcB3 LL, present in PE A4 and A14 strains, (C) FeoA and FeoB, present in PE A4 and A14 strains, and (D) ammonium transporter genes in PE A1 (*amtB1*), PE A4/A14 (*amtB1* LL), and duplication (*amtB2* LL) in PE A4/A14**. All panels show downwelling irradiance (μmol photons m^−2^s^−1^) measured at Mushroom Spring from September 11–12, 2009.

Transcript abundances for genes encoding components of the photosynthetic apparatus in the Mushroom Spring mat *Synechococcus* generally rise sharply at sunrise, are maximal during the mid-day, and decline in the late afternoon (see Figure 4D in Liu et al., 2012). This pattern is observed for *psbC*, which encodes a core subunit of the Photosystem II reaction center (Figure 2A). However, the transcript abundance for *isiX* has a different pattern and is most abundant during the low-light periods in the early morning (07:00–10:00) and late afternoon (15:00–19:00) (Figure 2A). Transcripts for the *apcD4* and *apcB3* genes have a similar overall abundance pattern to *isiX* (Figure 2B). Although transcripts for *apcA, apcB, apcD4*, and *apcB3* were all maximal at the same time in the morning (09:00), transcripts for *apcD4* and *apcB3* were maximal about an hour later than transcripts for *apcA* and *apcB* in the late afternoon period. Our confidence in the observed patterns is based on trends established by adjacent, closely spaced time points, highly similar co-expression patterns for different genes in this cassette, and correspondence with previously published transcription patterns (Steunou et al., 2006, 2008; Liu et al., 2012).

Another gene cassette unique to the low-light-adapted strains representative of PEs A4 and A14 contains the *feoAB* genes, which encode subunits of a ferrous iron transporter. The transcript abundances for these genes are maximal in the late afternoon, when the mat is becoming anoxic (Figure 2C; and see Figure 8C in Becraft et al., 2015). The most closely related FeoAB protein sequences are found in other cyanobacteria, but it is not clear if the genes were acquired by horizontal gene transfer, or lost in the high-light-adapted strains, which are representative of a PE that predominates in the more oxic portions of the mat. Strains of PEs A14 and A4 also share an ABC transporter cassette for sugar (possibly maltose/maltodextrin) transport and a paralogous methyl-accepting chemotaxis protein, one of many copies found in all four genomes. The latter gene has two identical in-frame stop codons in genomes of the high-light-adapted PE A1-OS and A1-MS strains.

In addition to horizontal gene transfer, gene duplication and subsequent nucleotide divergence can provide novel functionality to an organism, even though the resulting variant protein retains homology to the original product. All four strains contain a gene (*amtB1*) encoding a putative ammonium transporter, and the predicted AmtB1 proteins are ~90% identical. However, the strains of PEs A4 and A14 additionally contain a paralogous gene that has apparently arisen by duplication and divergence: AmtB2 is ~70% identical to AmtB1. The transcription patterns of the *amtB1* gene in the PE A1 strains and in the PE A4 and A14 strains are comparable, but the transcription pattern of the *amtB2* gene in the PE A4 and A14 strains differ from the *amtB1* pattern. This gene has a transcription pattern similar to many genes for components of the photosynthetic apparatus (Liu et al., 2012) and largely reflects the light period except for a late-afternoon decline (Figure 2D). Functional studies have shown that AmtB can transport both NH_3_ and CO_2_ (Musa-Aziz et al., 2009), and it is possible that these variants are functionally differentiated with respect to substrate. Because the transcript abundance pattern mirrors photosynthetic activity in the mat, this pattern is consistent with the possibility that AmtB2 could be a CO_2_ transporter. Alternatively, AmtB2 could transport ammonium but have a high affinity for the substrate. In addition to the duplicated *amtB* genes, strains of PEs A4 and A14 contain a second copy of *narB*, encoding assimilatory nitrate reductase, which is transcribed diurnally (Supplementary Figure S1). This copy is unlinked and divergent (~68% amino acid identity) from the nitrate reductase of the *nirA*-*narB* gene cluster found in all of the strains. Although this gene is also found in the genomes of the PE A1-MS and A1-OS strains, it is disrupted by a mobile element gene and thus is not likely to be active.

### Genes found only in high-light-adapted strains and their diel transcription patterns

The genomes of high-light-adapted strains PE A1-MS and A1-OS possess a copy of a carbonic anhydrase gene that has 68% amino acid identity to the zinc-dependent, gamma-class carbonic anhydrase found in *Thermosynechococcus* sp. NK55a. Carbonic anhydrase catalyzes the interconversion of carbon dioxide and bicarbonate by a reversible hydration reaction, and while the carbon-concentrating mechanism (CCM, present in all four strains) also includes a different carbonic anhydrase, cyanobacteria (Cannon et al., 2010), and other prokaryotes (Smith and Ferry, 2000) can have multiple copies of the genes and multiple classes of the enzyme that may play different functional roles in photosynthesis. The expression of this gene was too low to ascertain its transcription pattern confidently.

The urease cassette (Cluster 1 urease in Bhaya et al., 2007) found in the genome of the PE A1-OS strain is also found in the PE A1-MS strain genome, but these genes are not present in the genomes of the low-light adapted PE A4 and A14 strains. This urease cassette includes the genes that encode the larger alpha subunit UreC, smaller beta and gamma subunits UreB and UreA, which form the heterotrimeric urease enzyme, and UreDEFG accessory proteins that aid in assembly of the nickel metallocenter of the enzyme (Farrugia et al., 2013). All of the genes in the urease cassette have >90% identity to the urease genes found in *Thermus islandicus*, which indicates a possible horizontal gene transfer of this cassette to an ancestor common to the high-light-adapted strains but not the low-light-adapted strains.

The PE A1-OS and A1-MS strain genomes have a five-gene cluster annotated as a peptide/opine/nickel ABC transporter (PepT family), which includes a periplasmic substrate-binding protein, two permease subunits, and two ATP-binding protein genes. Additionally, the PE A1-OS and A1-MS strain genomes possess two components of a cystine ABC transporter, genes encoding the periplasmic cystine binding protein and the permease protein, as well as two genes, flanked by genes for mobile element proteins, that are annotated as succinate dehydrogenase flavoprotein subunit *sdhA* and omega-amino acid-pyruvate aminotransferase. Transcript abundances for all of these genes are higher during the light period and lower at night, similar to other genes that are expressed during the day (Supplementary Figure S2). Finally, along with the Type I and Type II CRISPR/*cas* arrays that are conserved among all four strains, the PE A1-MS genome contains a Type III CRISPR/*cas* array previously found in the PE A1-OS strain genome by Heidelberg et al. (2009). This is a unique CRISPR/*cas* array that is shared by *Roseiflexus* sp. RS-1, an anoxygenic photosynthetic organism that is also abundant in these microbial mat communities (Klatt et al., 2011). Although the amino acid similarities of the homologous genes are only 40–66% between the two organisms, there are transposons flanking the array in the PE A1 strain genomes, which suggests a possible, if not recent, lateral gene transfer event in the mat (Heidelberg et al., 2009).

### PsbA allele and diel transcription differences between high-light- and low-light-adapted strains

**Figure 3:**
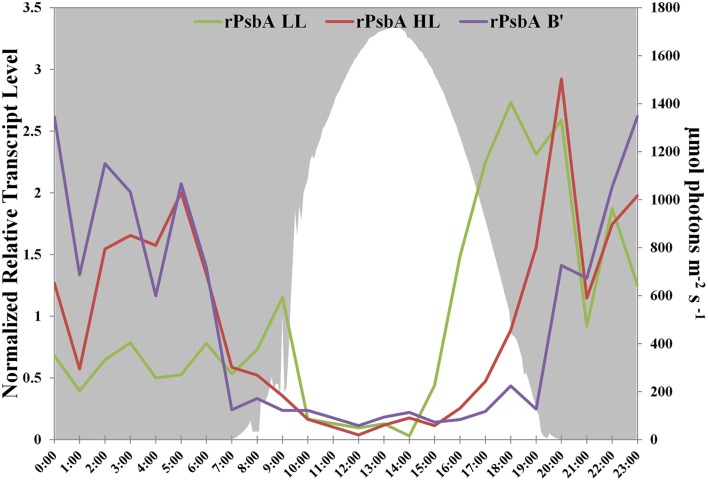
**rPsbA diel transcription patterns of alleles from the (i) B′ lineage, (ii) populations represented by high-light adapted strains (HL) of PE A1-OS and A1-MS, and (iii) populations represented by low-light adapted strains (LL) of PEs A4 and A14**. Downwelling irradiance (μmol photons m^−2^s^−1^) measured at Mushroom Spring on September 11–12, 2009, is also shown.

PsbA, also known as D1, is one of the core subunits of Photosystem II reaction center (Umena et al., 2011; Murray, 2012). The genome of the high-light-adapted PE A1-OS strain encodes four *psbA* genes, CYA_1274, CYA_1748, CYA_1811, and CYA_1894, while the B′ genome [JA-2-3B′a (2-13)] of *Synechococcus* has three *psbA* genes, designated CYB_0216, CYB_0371, and CYB_0433 (Bhaya et al., 2007). CYA_1274, CYA_1811, CYA_1849, CYB_0371, and CYB_0433 are nearly identical and differ by only 1 or 2 conserved amino acids. CYA_1748 and CYB_0216 are very similar to one another (94% identity, 96% similarity) but are only about 73% identical and 85% similar to the other PsbA sequences. These latter sequences have been called “rogue PsbA” sequences (rPsbA) by Murray (2012). Rogue PsbA sequences lack key functional residues and thus are not expected to support oxygen evolution by Photosystem II complexes that might contain them. The new strain genomes also possess multiple copies of the *psbA* gene: the PE A1-MS genome has four copies, which appear to be orthologous to those in the PE A1-OS genome, while the PE A4 and A14 genomes each have three *psbA* genes, two of which are identical to CYA_1274 and CYA_1849, as well as a copy of the *rpsbA* gene. The gene encoding rPsbA, CYA_1748, is sufficiently divergent to differentiate between the high-light- and low-light-adapted strains (the PE A4 and A14 alleles are 90 and 91% identical to the *rpsbA* gene in the PE A1-MS strain, respectively). Interestingly, the low-light- and high-light-adapted strains exhibited similar, but clearly temporally offset transcript abundance patterns (Figure 3), with the transcripts of low-light-adapted strains declining later in the morning and increasing earlier in the late afternoon. The transcript abundance for *rpsbA* (CYB_0216) of the B′-lineage *Synechococcus* also declines earlier in the morning than those of the low-light adapted strains, but they increase even later than transcripts of PE A1 strains.

### Other gene content differences among strains

The genomes of strains PEs A1-MS, A4, and A14 encode the genes necessary to synthesize urea carboxylase, including urea carboxylase/allophanate hydrolase, two urea carboxylase-related aminomethyltransferases, three genes for a urea carboxylase-related ABC transporter, and a biotin-protein ligase gene (Table 3). The urea carboxylase cluster proteins have between 60 and 85% identity to proteins found in other cyanobacteria, so it is not clear if the cluster was acquired horizontally or vertically. Strains of PEs A1-OS, A1-MS, and A4 share a three-gene cassette for an ABC transporter for polar amino acids that is not found in the PE A14 strain (Table 3). This is a potentially interesting difference because aspartate and glutamate are the only two amino acids that are not taken up and metabolized by *Chloracidobacterium thermophilum*, which is co-localized with the low-light-adapted *Synechococcus* that occur deeper in the mat (see Tank and Bryant, 2015). The PE A1-MS strain also has a gene cluster that is not found in the PE A1-OS strain and that consists of genes predicted to encode a bipolar DNA helicase, a Type I restriction-modification system DNA-methyltransferase subunit M, and the single-stranded exonuclease associated with Rad50/Mre11 complex (Table 3). The PE A14 strain possesses a PotABCD cassette for spermidine/putrescine transport that is not found in any of the other strains, which could provide an alternative nitrogen source for this strain. The proteins are most similar to PotABCD proteins in alpha and gamma proteobacteria, which may be indicative of a lateral gene transfer event. Additionally, the genome of the PE A14 strain encodes beta-carotene ketolase (CrtO), which is also encoded in the genome of the B′ strain JA-2-3B′a (2-13) (Bhaya et al., 2007). Keto-carotenoids provide better protection from reactive oxygen species than hydroxylated xanthophyll derivatives and are differently localized in membranes than other xanthophyll derivatives (Zhu et al., 2010). Further suggesting functional differences among these ecological species, transcripts were found for all of the strain-specific genes *in situ*.

## Discussion

In this study we compared the genomes of strains from extremely closely related yet ecologically distinct PEs, each of which has a unique distribution along the vertical gradient at 60–63°C (Becraft et al., 2015) and differing light adaptations and acclimation responses corresponding to their vertical distributions (Nowack et al., 2015). Our aim was to discover the genetic bases for the physiological differences that cause these organisms to occupy different niches along the vertical gradient. Some of the most conspicuous differences are found between the high-light-adapted strains of PE A1 and the low-light-adapted strains of PEs A4 and A14. Becraft et al. (2015), the first paper of this series, showed that PE A1 predominates in the upper to middle part (0–760 μm deep) of the upper green layer of the mat, while PEs A4 and A14 are most abundant in deeper layers of the mat (640–720 μm and 640–960 μm, respectively). The difference in scalar irradiance received by the different populations is striking (see Figure 8C in Becraft et al., 2015); while members of PE A1 may experience up to 1250 μmol photons m^−2^s^−1^ scalar irradiance, PE A4 and A14 populations may only experience 50–75 μmol photons m^−2^s^−1^ at the peak irradiance level during a diel cycle. These ecophysiological differences are reflected in the gene contents of these organisms.

Gene content differences suggest different adaptations for the high-light- and low-light-adapted organisms. The low-light-adapted strains of PEs A4 and A14 possess a gene cluster with xenologous copies of *apcD4, apcB3*, and *isiX*, all of which are highly expressed *in situ*. Genes in this cassette are most likely responsible for the long-wave absorption and fluorescence emission features observed in those strains when grown at low irradiance, but are missing in high-light-adapted organisms, as reported in the second paper of this series (Nowack et al., 2015). This would be consistent with selection pressure to improve and expand light harvesting when the ambient light is strongly filtered by Chl *a* and phycobiliproteins by organisms in the upper regions of the mat and by the greater relative abundance of far-red light at increasing depth in the mat (see Figure 4 in Becraft et al., 2015). Some of this shift to the far-red would simply be due to greater penetration by light of longer wavelengths, which is less readily scattered. This gene cassette is also found in several other cyanobacteria (see Shih et al., 2013 Figure S5 CP43 phylogeny, members of clade CBPVI; and Gan et al., 2014b), including *Chlorogloeopsis* spp. PCC 6912 and PCC 9212, *Fischerella* sp. PCC 9605, *Chroococcidiopsis thermalis* PCC 7203, *Gloeocapsa* sp. PCC 7428, *Xenococcus* sp. PCC 7305, and *Leptolyngbya* sp. PCC 6406. These genes may encode a common adaptive mechanism among low-light-adapted cyanobacteria that are primarily found in benthic or terrestrial environments, by enabling them to acclimate to low irradiance conditions and/or to far-red light. Only three genes, *apcD4, apcB3*, and *isiX*, are required, which is far simpler than the FaRLiP response recently described by Gan et al. (2014a) that involves 17 genes and leads to changes to all three major photosynthetic complexes. Interestingly, several organisms that can perform FaRLiP (Gan et al., 2014a,b) also have this simpler system, which strongly suggests that the systems, at least in those cyanobacteria that have both capabilities, respond to different light cues.

The PE A4 and A14 strains additionally contain the *feoAB* genes for the ferrous iron transport system, which were initially described to be present in the metagenomes of the Mushroom Spring and Octopus Spring mats. The transcription pattern of the genes in the metatranscriptome (Liu et al., 2012 and Figure 2C) match the transcription pattern of *feoB* measured with q-RT-PCR over a diel cycle by Bhaya et al. (2007). Under alkaline conditions, ferrous iron is only present in the absence of oxygen, which may occur more often in the deeper parts of the mat, away from the higher levels of oxygen in the upper part of the mat that are produced by *Synechococcus* populations experiencing higher irradiance levels and longer periods of exposure to light (Jensen et al., 2010). Interestingly, the *feoAB* genes discovered by Bhaya et al. (2007) were found on metagenomic clones that were most closely related to the B′-lineage, which may indicate the existence of low-light-adapted B′-lineage ecotypes as well as low-light-adapted A-lineage PEs. This might explain the inability of the B′-like strain studied by Kilian et al. (2007) to grow at high irradiance, if it contained only low-light-adapted, B′-lineage ecotypes.

In contrast to the low-light-adapted strains, the high-light-adapted strains PE A1-OS and A1-MS both contain an extra carbonic anhydrase gene, which may enhance growth under CO_2_-limiting conditions when bicarbonate is present. The extra carbonic anhydrase may enhance conversion of bicarbonate to CO_2_. CO_2_ limitation caused by high rates of photosynthesis during peak irradiance has been indicated by an increase in pH when rates of oxygenic photosynthesis are high (Jensen et al., 2010). This observation led us to demonstrate that the growth rate of the PE A1-MS strain, but not a strain without the extra carbonic anhydrase gene, was increased by the addition of bicarbonate under carbon-limiting conditions (Supplementary Figure S3 and Supplementary Methods), which implies that this gene may provide increased fitness under such conditions. The high-light-adapted PE A1-OS and A1-MS strains also have unique genes involved in the TCA cycle (*sdhA*) and virus infection (Type III CRISPR/*cas* array), which may be indicative of uncharacterized environmental realities of the high-light-adapted strains compared to the low-light-adapted strains.

Transcription patterns differ for genes associated with strains representative of different PEs. We were able to exploit the relatively high sequence divergence of the *rpsbA* gene to show that the transcription timing of this gene by low-light-adapted PE A4 and A14 populations found deepest in the mat green layer differed from that of the high-light-adapted PE A1 population residing above them. Specifically, transcription in PEs found deeper in the mat started earlier in the afternoon and ended later in the morning. Jensen et al. (2010) reported a similar transcription pattern for this gene in B′-like *Synechococcus*. Furthermore, by recruiting B′-like transcripts from the metatranscriptome, we were able to show that B′-like populations in the 60°C mat, which have been shown to predominate in the uppermost part of the mat green layer (see Figure 3 of Becraft et al., 2015 and Ramsing et al., 2000), express *rpsbA* genes even later in the afternoon and have declining transcript abundances for these genes even earlier in the morning. Similarly, Becraft et al. (2015) reported offsets in the timing of B′-like and A-like expression of photosynthesis and nitrogen fixation genes.

The function of rogue-PsbA in Photosystem II has not yet been established, but because this subunit is missing essential amino acid residues for the Mn_4_CaO_5_ cluster of the water oxidation center and has key differences in the binding pocket for quinone Q_B_, it seems unlikely that Photosystem II complexes containing this protein can oxidize water (Murray, 2012). Considering that transcript abundance pattern for this gene is similar to those for nitrogen fixation genes (Figure 8B in Becraft et al., 2015), and that transcripts for “typical” *psbA* alleles increase rapidly as nitrogen fixation wanes and photosynthesis increases, we hypothesize that rPsbA subunits are involved in the oxidation of sulfide, which is present in the mats due to sulfate reduction during periods of anoxia (van der Meer et al., 2005; Dillon et al., 2007). Although *Synechococcus* lacks sulfide quinone reductase, which occurs in some cyanobacteria that oxidize sulfide to polysulfide (e.g., *Oscillatoria limnetica*; Arieli et al., 1991, 1994), most cyanobacteria that oxidize sulfide actually produce thiosulfate as the sole product in a reaction that has never been fully characterized biochemically (De Wit and van Gemerden, 1987; Rabenstein et al., 1995). We hypothesize that rPsbA is involved in the oxidation of sulfide to thiosulfate, and that this process could provide electrons for nitrogen fixation by nitrogenase, which would otherwise be inactivated by oxygen production if Photosystem II contained “typical” PsbA subunits. This scenario is further supported and is completely consistent with previous results suggesting that sulfide stimulated early morning incorporation of CO_2_ into cyanobacterial lipids (van der Meer et al., 2005). Such a process would be expected to occur under anoxic conditions, which occur earlier in the afternoon in deeper portions of the mat (see Figure 8C in Becraft et al., 2015).

Additionally, we observed gene content differences among strains that might reflect alternative strategies for nitrogen metabolism. For instance, both PE A1 strains are capable of urea degradation with urease, while strains of PEs A1-MS, A4, and A14 have urea carboxylase. Urea degradation with urea carboxylase involves two separate reactions and is ATP-dependent, while urease involves only one reaction and is not ATP-dependent, but requires nickel for the enzyme metallocenter (Sakamoto and Bryant, 2001; Solomon et al., 2010; Farrugia et al., 2013). Rates of urea uptake are usually higher than for nitrate or nitrite, even when the concentration of these oxidized nitrogen sources is higher, and urea is preferable in CO_2_-limited environments because CO_2_ is a useful by-product of urea assimilation (Solomon et al., 2010). The peptide/opine/nickel transport cassette in PE A1-OS and A1-MS strains may provide the nickel for the urease enzyme when available, or it might be involved in scavenging of environmental peptides or opines as a source of both nitrogen and organic carbon. Similarly, the cystine transport genes in PE A1-OS and A1-MS strains, the polar amino acid transport cassette in PE A1-OS, A1-MS, and A4 strains, and the PotABCD spermidine/putrescine transporter in the PE A14 strain are all transcribed *in situ*, and all transport possible sources of nitrogen into the cells. Other gene content differences among strains may indicate differences in organic carbon use (the putative MalK transport cassette in strains of PEs A4 and A14) and DNA protection and repair (bipolar DNA helicase and single-strand exonuclease in the PE A1-MS strain and beta-carotene ketolase in the PE A14 strain). Some of these gene content differences may help to explain the niche differentiation between the two low-light-adapted strains of PEs A4 and A14. Although both grow faster at lower irradiances than the PE A1 strains and are thus characterized as low-light-adapted, they do have different patterns of growth relative to light intensity (Nowack et al., 2015) and different vertical distributions in the mat (Becraft et al., 2015). The PE A4 distribution is maximal in the lower-middle part of the mat upper green layer, while PE A14 is maximal at the greatest depths where irradiance is most attenuated.

This three-paper series was designed to address the issue of the molecular dimension of microbial species. Since Woese and Fox (1977) used the highly conserved 16S ribosomal RNA sequence to estimate phylogenetic relatedness among organisms to reveal inaccuracies of traditional classification methods [e.g., the complete oversight of the domain Archaea (Balch et al., 1977)], the extensive use of this approach has led to a somewhat arbitrary molecular demarcation of microbial species that is widely accepted and used by many microbiologists. Molecular cutoffs were created by observing the sequence divergence among strains of classically named species (e.g., Seki et al., 1978 within the genus *Bacillus*): that a >3% divergence of the 16S rRNA locus between two organisms (Stackebrandt and Goebel, 1994) or, more recently, >1% divergence at the 16S rRNA locus (Stackebrandt and Ebers, 2006) is required to consider that the two strains belong to different species. Using the highly resolving locus *psaA*, we have (i) predicted the existence of different putative ecological species within traditional 16S rRNA-defined species using a theory-based model (Becraft et al., 2011), (ii) shown that they are ecologically distinct through differences in distribution, (iii) shown that many contain ecologically homogeneous members (Becraft et al., 2015), (iv) shown that strains representative of different PEs have different adaptations and acclimation responses to light (Nowack et al., 2015), and (v) through comparative genomic analysis of these strains, shown that strains of the different PEs contain differences in gene content and gene alleles which appear to underlie the adaptations and acclimation responses of each PE to their distinct ecological niches. Comparative analyses of multiple strains of different PEs, including analyses of genes under selection within and between ecotypes, will provide further evidence for the ecological differentiation among PEs and will demonstrate whether the adaptations and acclimative responses of these strains are typical of members of a PE. These results, along with the differences in the timing of gene expression by different PEs located in distinct niches, demonstrate that extremely closely related ecologically adapted populations, which may in fact be true ecological species, matter in microbial communities.

## Author contributions

MO cultivated strains, performed molecular procedures, performed genomic analyses, and wrote the manuscript. SN cultivated strains and performed growth experiments. JW and EB assisted in analyzing sequence data. EB assisted with molecular procedures. KL, AL, JM, and WS assisted in genome sequencing and assembly. DR, FC, and DB were acting Co-PIs for the Joint Genome Institute Community sequencing project. FC and DB discussed results and edited the manuscript. DW was acting PI for the Joint Genome Institute Community sequencing project, assisted with experimental design, discussed the results, and edited the manuscript at all stages.

### Conflict of interest statement

The authors declare that the research was conducted in the absence of any commercial or financial relationships that could be construed as a potential conflict of interest.

## Footnotes

^1^http://www.rcn.montana.edu/Features/DataDetail.aspx?id=124 for Mushroom Spring data and http://www.rcn.montana.edu/Features/DataDetail.aspx?id=37 for Octopus Spring data

^2^http://www.jgi.doe.gov/
