# Supplementary figures and images for: The molecular dimension of microbial species: 3. Comparative genomics of Synechococcus strains with different light responses and in situ diel transcription patterns of associated putative ecotypes in the Mushroom Spring microbial mat
Source: Front Microbiol. 2015 Jun 23;6:604. doi: 10.3389/fmicb.2015.00604 (PMC4477158; doi:10.3389/fmicb.2015.00604)

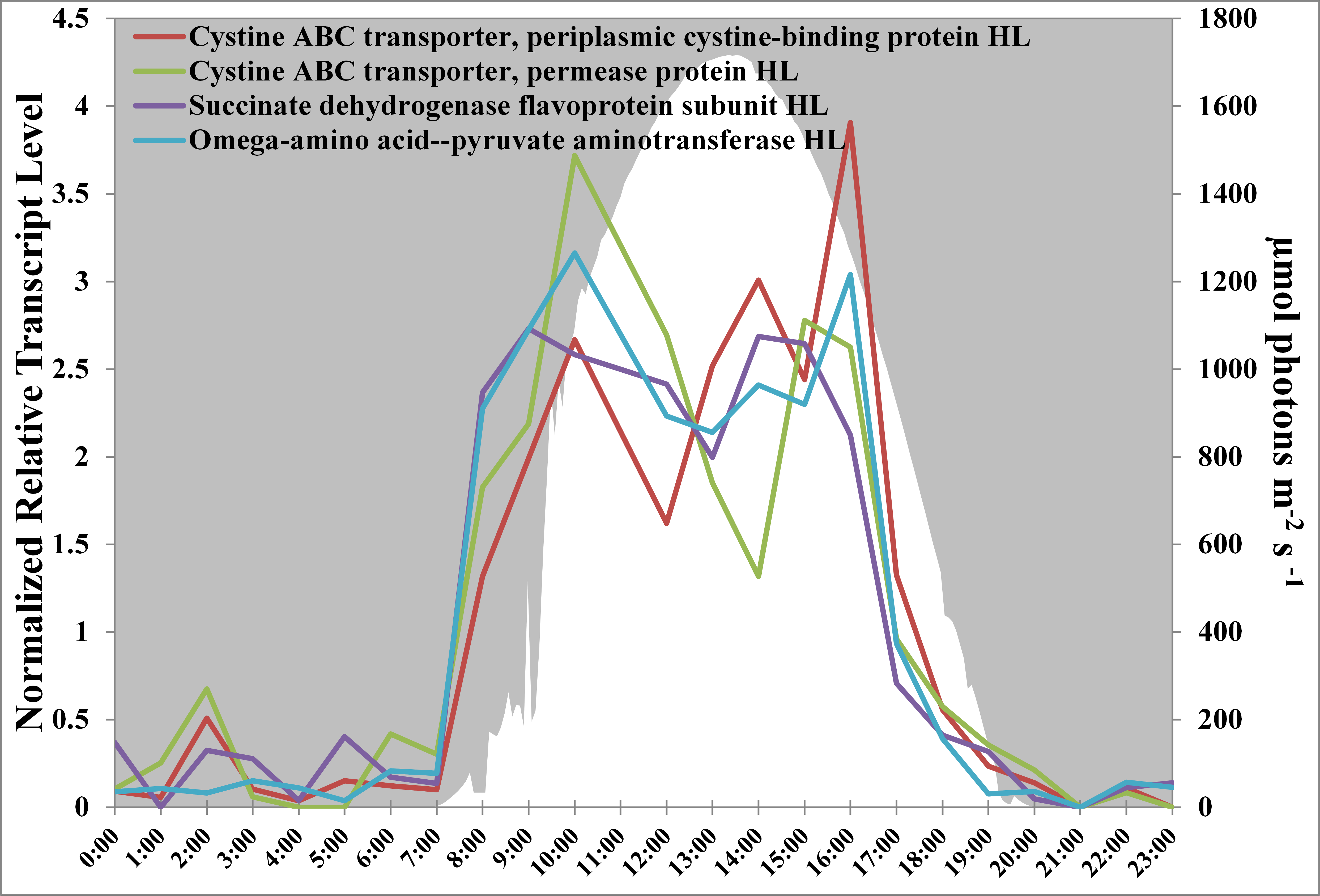

Supplement: Supplementary file 1 [file Image1.TIF]

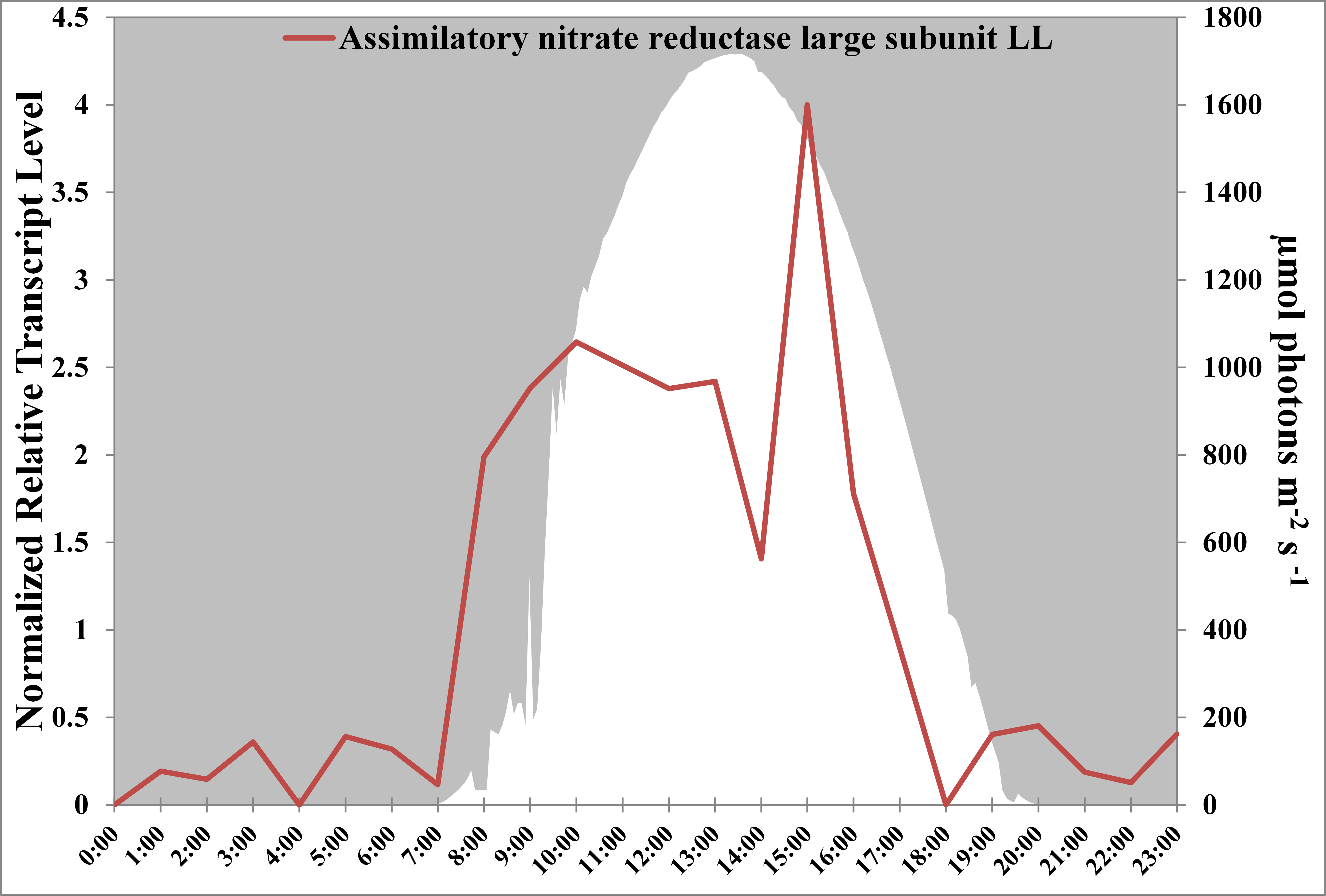

Supplement: Supplementary file 2 [file Image2.TIF]

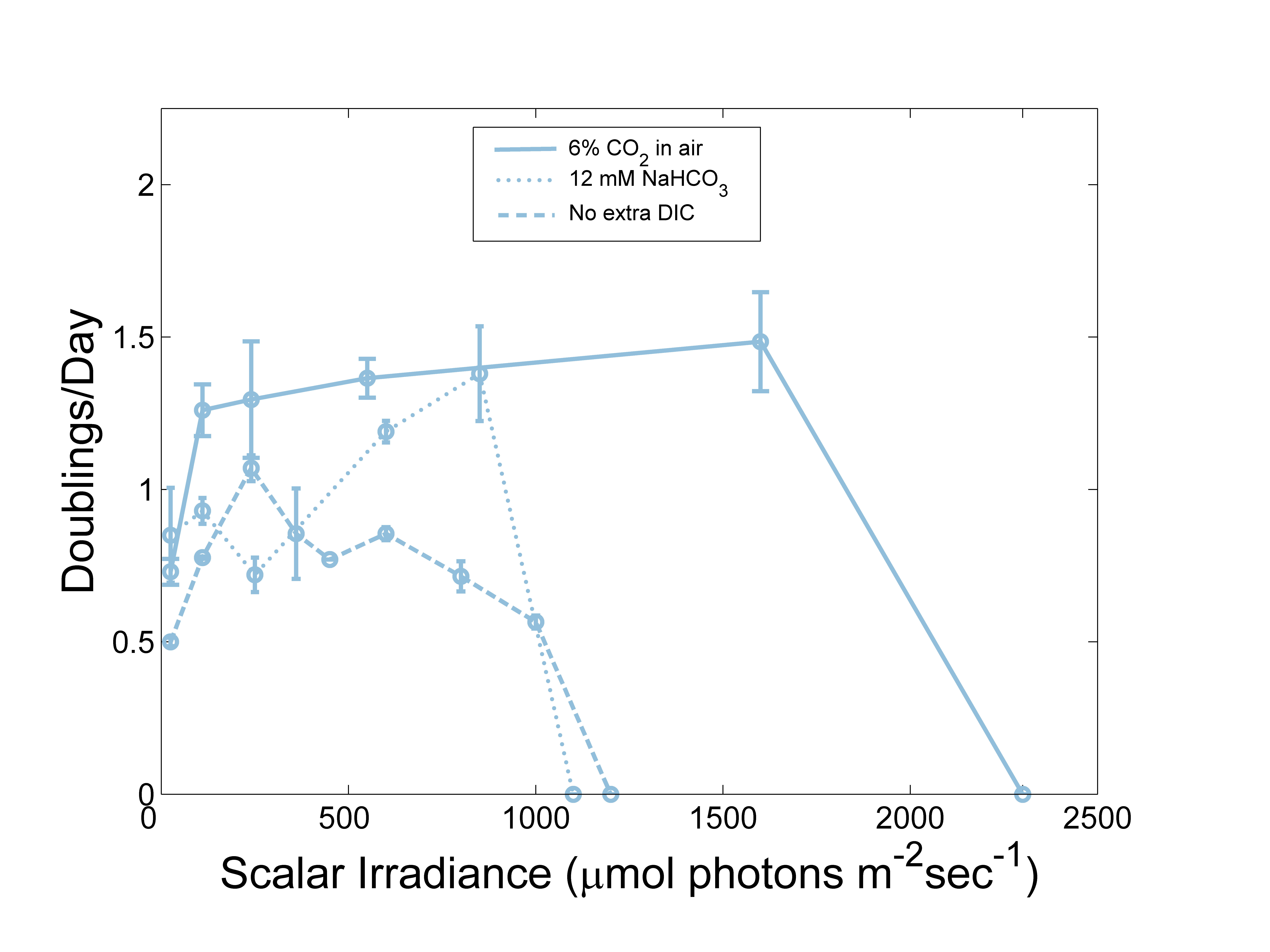

Supplement: Supplementary file 3 [file Image3.TIF]
