# Supplementary material for: The molecular dimension of microbial species: 3. Comparative genomics of Synechococcus strains with different light responses and in situ diel transcription patterns of associated putative ecotypes in the Mushroom Spring microbial mat
Source: Front Microbiol. 2015 Jun 23;6:604. doi: 10.3389/fmicb.2015.00604 (PMC4477158; doi:10.3389/fmicb.2015.00604)
Supplement: Supplementary file 4 [file DataSheet1.DOCX]

***Supplementary Material***

**Comparative genomics of *Synechococcus* isolates with different light responses and *in* *situ* diel transcription patterns of associated putative ecotypes in the Mushroom Spring microbial mat**

**Millie T. Olsen^1*^, Shane Nowack^2^, Jason M. Wood^1^, Eric D. Becraft^1^, Kurt LaButti^3^, Anna Lipzen^3^, Joel Martin^3^, Wendy S. Schackwitz^3^, Douglas B. Rusch^4^, Frederick M. Cohan^5^, Donald A. Bryant^6, 7^, and David M. Ward^1^**

^1^Department of Land Resources and Environmental Sciences, Montana State University, Bozeman, MT, USA

^2^Department of Mathematical Sciences, Montana State University, Bozeman, MT, USA

^3^Department of Energy, Joint Genome Institute, Walnut Creek, CA, USA

^4^J. Craig Venter Institute, Rockville, MD, USA

^5^Department of Biology, Weslyan University, Middletown, CT, USA

^6^Department of Biochemistry and Molecular Biology, The Pennsylvania State University, University Park, PA, USA

^7^Department of Chemistry and Biochemistry, Montana State University, Bozeman, MT, USA

*** Correspondence:** Millie T. Olsen, Department of Land Resources and Environmental Sciences, Montana State University, 960 Technology Blvd, Bozeman, MT, 59718,USA.

millie.thornton@msu.montana.edu

1. **Supplementary Data**

**Stimulation of *Synechococcus* isolate A1-MS by bicarbonate**

To assess the interconnectivity between light responses and the form of available dissolved inorganic carbon (DIC), the growth rates with respect to light intensity of the *Synechococcus* PE A1-MS isolate were measured when three different forms of DIC were provided. The genome sequence of this isolate contains an extra putative carbonic anhydrase gene that the low-light-adapted isolates do not possess. This gene is known to produce an enzyme that catalyzes the bi-directional interconversion of CO_2_ and water to bicarbonate. Hence, we hypothesized that the PE A1-MS isolate would be stimulated by addition of bicarbonate under conditions of CO_2_ limitation. The protocol to measure growth rates described in Nowack et al. (this issue) was followed with these additional specifications. The cultures were pre-grown at 52°C and at a scalar irradiance of 50 μmol photons m^–2^s^–1^, and the temperature during the growth rate experiments was 52°C. To alleviate CO_2_ limitation in one set of conditions, 6% CO_2_ in air was sparged at a rate of ~ 1 bubble/second. To achieve conditions of CO_2_ limitation, the only form of DIC was diffusion from the air above the culture. Filter-sterilized NaHCO_3_ was added (to 12 mM final concentration) to provide conditions in which bicarbonate was the primary DIC source. Bicarbonate stimulated the rate of growth of this isolate at irradiances typically observed at the depth in the mat at which PE A1 is the predominant *Synechococcus* population (Figure S3 and see Figures 3 and 8C in Becraft et al., this issue).

1. **Supplementary Figures and Tables**

**Figure S1. PE A1-specific genes:** Diel transcription patterns of PE A1-specific cystine ABC transporter periplasmic binding protein and permease protein, succinate dehydrogenase flavoprotein subunit *sdhA*, and omega-amino-acid-pyruvate transferase with downwelling irradiance (µmol photons m^–2^s^–1^) measured at Mushroom Spring from September 11-12, 2009.

**Figure S2. Diel transcription patterns of assimilatory nitrate reductase duplicate copy in PEs A4 and A14 with downwelling irradiance (µmol photons m^–2^s^–1^) measured at Mushroom Spring from September 11-12, 2009.**

**Figure S3. Growth of the *Synechococcus* isolate of PE A1-MS strain as a function of light intensity and differences in forms of dissolved inorganic carbon supplied.**

**Table S1. Orthologous genes among *Synechococcus* isolates**. Columns represent gene locus number, percent amino acid identity to the A1-OS reference protein (when applicable), and RAST annotated gene name for each isolate, as well as the designated Phyla-AMPHORA marker peptide name. Genes not present in all four isolates are highlighted in green, while gene absence in an isolate is highlighted in yellow. Genes highlighted in orange have >90% amino acid identity to the PE A1-OS isolate reference protein sequence. Phyla-AMPHORA marker proteins are highlighted in blue. Any manual verification of gene absence is noted in red text within the yellow highlighted cell.
